# Nuclear dynamics and stress responses in Alzheimer’s disease

**DOI:** 10.1186/s13024-021-00489-6

**Published:** 2021-09-17

**Authors:** Artemis Iatrou, Eric M. Clark, Yanling Wang

**Affiliations:** Rush Alzheimer’s Disease Center, Rush University Medical Center, 1750 W. Harrison St., Chicago, IL 60612 USA

**Keywords:** Nucleus, Alzheimer’s disease, Chromatin, Gene regulations, Cell cycle deregulation

## Abstract

In response to extracellular and intracellular stressors, the nucleus and nuclear compartments undergo distinct molecular changes to maintain cell homeostasis. In the context of Alzheimer’s disease, misfolded proteins and various cellular stressors lead to profound structural and molecular changes at the nucleus. This review summarizes recent research on nuclear alterations in AD development, from the nuclear envelope changes to chromatin and epigenetic regulation and then to common nuclear stress responses. Finally, we provide our thoughts on the importance of understanding cell-type-specific changes and identifying upstream causal events in AD pathogenesis and highlight novel sequencing and gene perturbation technologies to address those challenges.

## Background

The membrane-bound nucleus is one of the complex features that arose from prokaryotic to eukaryotic cells through evolution. It contains genetic materials and acts as the control center of the cell to control the synthesis of ribosomes and proteins. Under normal circumstances, the nucleus regulates gene expression to maintain cell homeostasis. In response to environmental and intracellular insults, cells relay the “stress signals” through various signaling pathways to the nucleus to defend cells against stress and restore homeostasis.

An emerging concept that unifies Alzheimer’s disease (AD) and other neurodegenerative diseases is that chronic response to oxidative stress and misfolded proteins disrupts neuronal function, leading eventually to neurodegeneration. In AD, cellular stress is often initiated by oxidative stress and further enhanced by neurotoxic amyloid-beta (Aβ) oligomers and phosphorylated tau (p-tau), as well as the release of inflammatory mediators [1]. With the nucleus being a point of convergence for stress response, a better understanding of its structural, molecular and functional changes would highlight intracellular underpinnings of AD pathogenic processes.

Accumulated studies have shown that cellular insults induce profound changes to the nuclear structure, as well as the epigenome and transcriptome in AD brains [2]. Here we summarize the recent literature on these nuclear changes in animal models of AD and AD postmortem brain tissue. We first outline the nuclear structure changes from the nuclear envelope and nuclear pore complexes (NPCs) to the nucleolus, then elaborate on multiple layers of epigenetic regulation of gene expression. Furthermore, we discuss DNA damage response (DDR) and cell cycle deregulation in AD pathogenesis. Lastly, we provide our thoughts on refining the molecular signature of AD and identifying the causal genes for therapeutic intervention.

## Main text

### Nuclear envelope and nucleolus in AD

#### Nuclear envelope in AD

**Fig. 1:**
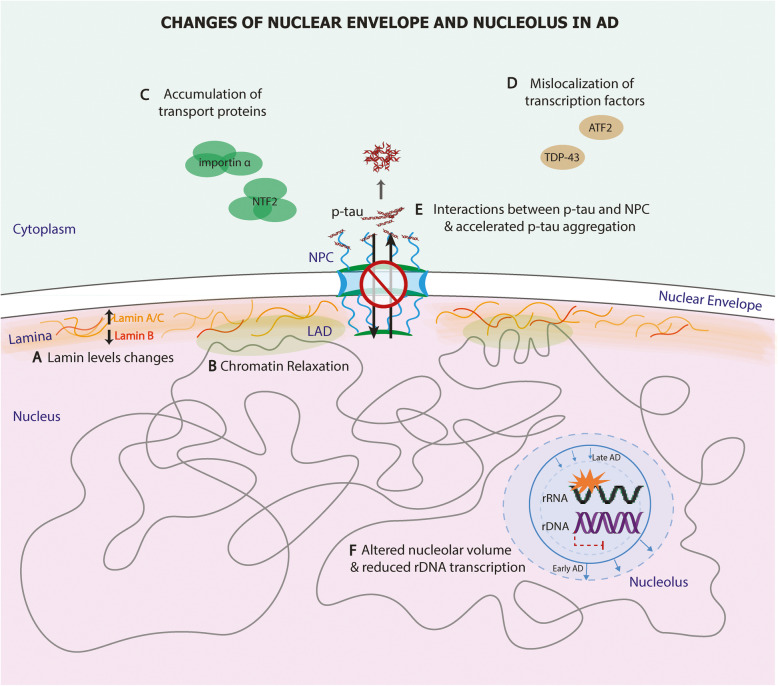
Nuclear envelope and nucleolus changes in AD. **A** In the nuclear lamina, lamin A/C expression is increased, whereas lamin B is reduced in the AD cortex. **B** Dysfunctional lamina causes pathological chromatin relaxation at lamina-associated domains (LAD). **C** Abnormal accumulation of nuclear pore complex (NPC)-associated proteins and other nuclear transport factors, i.e. NTF2 and importin α, compromises nucleocytoplasmic transport. **D** Various transcription factors are found mislocalized to the cytoplasm. **E** NPC components are found mislocalized to the cytoplasm, interacting with neurofibrillary tangles (NFTs), leading to accelerated phosphorylated tau aggregation and eventually impaired nucleoplasmic transport. **F** The volume of the nucleolus increases at the early stage of AD but decreases as AD progresses. In the nucleolus, ribosomal DNA (rDNA) transcription reduces, and ribosomal RNA (rRNA) is damaged by oxidative stress

The nuclear envelope is a highly dynamic structure, consisting of the nuclear lamina and a double membrane connected at specific points where the NPCs form [3]. The nuclear lamina forms a dense fibrillar network regulating important cellular events such as DNA replication, gene regulation, and signal transduction. Lamins, the major architectural proteins of the lamina, also serve as a scaffold to tether chromatin-protein complexes to the nuclear lamina, thereby sustaining genomic stability. Lamina-associated domains (LADs), the chromatin positioned close to the nuclear lamina, display typical heterochromatin features and are usually flanked by insulator protein CTCF-binding sites [4]. Increased lamin A and lamin C levels have been detected with the aggravation of AD pathology in postmortem hippocampus [5], whereas lamin B levels are reduced in AD frontal cortices (Fig. 1A) [6]. In the same study, lamin dysfunction in a tau-transgenic *Drosophila melanogaster* AD model led to heterochromatin relaxation (Fig. 1B), DNA damage, and neuronal degeneration [6]. Interestingly, pharmacologic and genetic inhibition of thioredoxin1, an antioxidant, enhanced caspase-6 activity in serum-deprived SH-SY5Y neurons, which resulted in the degradation of lamin B1 and nuclear envelop invagination [7]. This study indicates that thioredoxin1 a key regulator for nuclear lamina integrity. Consistently, reduced thioredoxin1 was detected in AD mouse brain, a finding also reported in AD postmortem brains [8].

The NPC is embedded in the nuclear envelope, containing more than 500 copies of 30 distinct nucleoporin proteins (Nups). NPCs mediate selective nucleocytoplasmic transport by forming a permeability barrier with the intrinsically disordered phenylalanine-glycine-rich Nups (FG-Nups) in the center and scaffold Nups in the periphery [9]. Nups also play an essential role in transcriptional regulation to determine cellular fate and identity of various cell types in the brain [10], likely through coordinating super-enhancers [11].

Multiple studies have shown that the NPC structure and nucleocytoplasmic transport are altered in AD. Initial evidence came from the immunolabeling of nucleoporins and NPC-associated proteins on postmortem hippocampal sections. This study demonstrated increased nuclear irregularity accompanying intracellular neurofibrillary tangles (NFTs) in AD hippocampal neurons [12]. They also observed abnormal perinuclear accumulation of nuclear transport factor 2 (NTF2), a critical NPC-associated protein, in scattered CA1 neurons in AD (Fig. 1C). Furthermore, importin α, an essential protein of cytoplasmic-nuclear transport, was also found accumulated in human AD hippocampal CA1 neurons [13] (Fig. 1C). Lastly, various transcription factors, such as TDP-43 [14] and ATF2 [14], were found mislocalized to the cytoplasm of AD neurons (Fig. 1D). These studies indicate the dysfunction of nucleocytoplasmic transport in AD. A recent study provided further evidence that pathological tau directly interacts with components of the NPC, including Nup98, leading to accelerated tau aggregation in the cytoplasm and impaired nucleocytoplasmic transport [15] (Fig. 1E). As expected, reducing soluble p-tau and Nup98 can restore nucleocytoplasmic transport in rTg4510 mice [15]. Concordantly, Paonessa et al. showed that tau mutations resulted in its hyperphosphorylation and mislocalization from axons to cell bodies and dendrites in stem cell-derived neurons, leading to nuclear membrane deformation and nucleocytoplasmic transport defect [14].

#### Nucleolus in AD

The nucleolus, consisting of ribosomal DNA (rDNA), ribosomal RNA (rRNA), and proteins, is the site for ribosome biogenesis [16]. The nucleolus is compartmentalized into the fibrillar center, the dense fibrillar center, and the granular component for pre-rRNA transcription, processing, and ribosomal ribonucleoprotein (RNP) assembly respectively [17, 18]. Ribosomal biogenesis requires 80% of the cellular energy; therefore, cellular metabolism can directly affect nucleolar activities.

Abnormal nucleoli morphology and function have been implicated in AD [19]. Using design-based stereology, Iacono et al. measured the volumes of neuronal cell bodies, nuclei, and nucleoli in postmortem cortex and hippocampus [20, 21]. Interestingly, asymptomatic AD demonstrated significant neuronal hypertrophy, especially profound nucleoli hypertrophy in CA1 neurons of the hippocampus, compared with mild cognitive impairment (MCI) cases with a similar load of AD pathology (Braak III-V) [20, 21], indicating a compensatory mechanism that prevents the disease progression into dementia. In contrast, definitive AD cases (Braak IV-VI) demonstrated significant atrophy of the neuronal cell bodies and nucleoli in the CA1 region [20, 21]. In line with this, Tagliavini et al. found significantly reduced nucleolar volume in the basal nucleus of Meynert, and the percentage volume reduction correlated with the percentage of cell loss in this region [20–22] (Fig. 1F).

Accumulated studies have indicated that rDNA transcription is regulated by different tau species. Immunogold labeling of human brain sections has shown that tau is expressed within the nucleolus and colocalizes with TIP5, a key player in heterochromatin stability, indicating a potential role for tau in rDNA transcriptional repression. Indeed, depleting tau in SH-SY5Y neuroblastoma cells decreases heterochromatin and DNA methylation, increasing rDNA transcription [23]. Federico et al. studied the cellular localization of the phosphorylated AT8 (Ser202/Thr205) and unphosphorylated Tau1 (Pro189/Gly207) epitopes of tau protein in the SK-N-BE cell line. They detected punctated staining for Tau1 in nucleoli of both proliferative and differentiated cells, whereas diffused AT8 staining in the entire nucleolus of only differentiated cells [24]. Since the transcriptional activity is reduced in differentiated cells, this study also supports a possible role of rDNA silencing for p-tau during neuronal differentiation. It has been reported that AD patients have hypermethylated rDNA promoters and reduced rDNA transcription [25] (Fig. 1F). Whether this reduced rDNA transcription results from tau or p-tau is yet to be studied. Nevertheless, nuclear tau species may function differently under cellular stress. For example, glutamate-induced cellular stress triggered the redistribution of nucleolar tau, but not p-tau [23]. Recently, Gil et al. conducted immunohistochemistry on postmortem brains at different ages and revealed that p-tau, AT100 (Thr212/Ser214), progressively increased in nuclei during aging and co-localized with the DAPI-positive heterochromatin [26]. Interestingly, AT100 was also detected in the nucleolus of pyramidal neurons in the CA1 region, with its highest expression in senescent cells in early AD stages and disappearing in more advanced stages [26] (Fig. 1F). In the same study pronounced AT100 expression in nucleoli at Braak stage I was in concordance with nucleolar hypertrophy while the absence of AT100 matched the drastic reduction in nucleolar volume observed in stages IV-V [26] (Fig. 1F).

In vitro culture and animal studies have also demonstrated the nucleolar responses to Aβ-related pathologies. Incubation of SH-SY5Y neuroblastoma cells with Αβ oligomers for 24 h altered distribution of nucleolar tau, induced nucleolar stress and a reduction of rRNA synthesis and protein production [27]. Garcia-Esparcia et al. conducted a comparative study on nucleolar and ribosomal molecules in the cortex of postmortem AD individuals (Braak stage V-VI) versus APP/PS1 mice, and they detected significant but divergent protein and gene alternations related to the protein synthesis machinery from the nucleolus to the ribosome [28]. Furthermore, a recent study identified a long nucleolus-specific lncRNA (LoNA) that can serve as a sensor of neuronal activities, and its activity-dependent decrease leads to elevated rRNA levels, ribosome biosynthesis, and protein translation [29]. Notably, LoNA expression was elevated in the hippocampus of APP/PS1 mice, accompanied by reduced levels of rRNAs, and knockdown of LoNA restored rRNA expression and rescued cognitive and memory impairments in the same AD mouse model [29].

Recent studies have attributed nucleolar stress response as a novel signaling pathway in early AD development (reviewed in [30]). For example, SHSY5Y cells treated with Aβ42 oligomers for 2 h showed oxidative stress and a significant reduction in UBF, a nucleolar transcription factor that drives the transcription of rDNA [30]. Furthermore, oxidative stress can directly affect rRNA, contributing to ribosome dysfunction by increasing the iron-binding capacity of rRNA. Consistent with this, ribosomes purified from the AD hippocampus contained significantly higher levels of RNase-sensitive iron and redox activity [31]. In addition, AD and MCI cortices demonstrated elevated rRNA oxidation and reduced rRNA level [32, 33] (Fig. 1D). Lastly, the application of DNA damage reagents or blocking rRNA synthesis reduced nucleolar rRNA transcription, leading to p53-dependent protracted neuronal degeneration in vitro [31, 34]. Therefore, the nucleolus may serve as a critical stress-sensor and gatekeeper to maintain the cell homeostatic state, initiating neurodegenerative molecular changes upon cellular stress.

### Nuclear chromatin in AD

#### Histone modifications in AD

Histone post-translational modifications (PTMs) are a significant contributor to the epigenetic regulation of gene expression. Histone methylation and histone acetylation are the two common but distinct forms of histone PTMs. Histone methylation, catalyzed by histone methyltransferases, occurs on specific N-terminus lysines of histones H3 and H4 to either increase or repress transcription of the nearby genes [35]. Histone acetylation, executed by histone acetyltransferases (HATs), generally results in transcriptional activation; conversely, histone deacetylases (HDACs) reverse histone acetylation and suppress transcription.

Histone methylation changes linked to heterochromatin state have been implicated in AD but remain inconclusive. Frost et al. examined the H3K9me2, a heterochromatin mark for constitutive telomeric and pericentromeric heterochromatin along with the heterochromatin protein 1α (HP1α) in tau-induced neurodegeneration [36]. They found widespread loss of these heterochromatin marks and aberrant gene expression in tau transgenic *Drosophila* and mice, and in the human AD hippocampus (Braak stages V/VI). Leveraging public chromatin immunoprecipitation followed by sequencing (ChIP-seq) datasets from human AD brains, they also revealed a widespread transcriptional increase in genes silenced in controls due to heterochromatin state [36]. On the contrary, Zheng and others used similar experimental approaches but detected significant elevation of H3K9me2 in 5XFAD mouse model and the prefrontal cortex of postmortem human AD brains. Concomitantly, H3K9me2 at glutamate receptors was increased in the prefrontal cortex of aged 5XFAD mice; treating FAD mice with specific histone methyltransferase inhibitors, reversed histone hypermethylation, restored glutamate receptor expression and cognitive impairment [37]. Meanwhile, Lee and others discovered that H3K9me3-mediated heterochromatin condensation was also elevated in sporadic AD postmortem cortices (Braak stages V/VI). By combining H3K9me3 ChIP-seq and mRNA-seq, they discovered that epigenomes highly occupied by H3K9me3 were inversely correlated with their mRNA expression levels in AD, and the downregulated genes were mainly involved in synaptic function and neuronal differentiation [38].

**Fig. 2:**
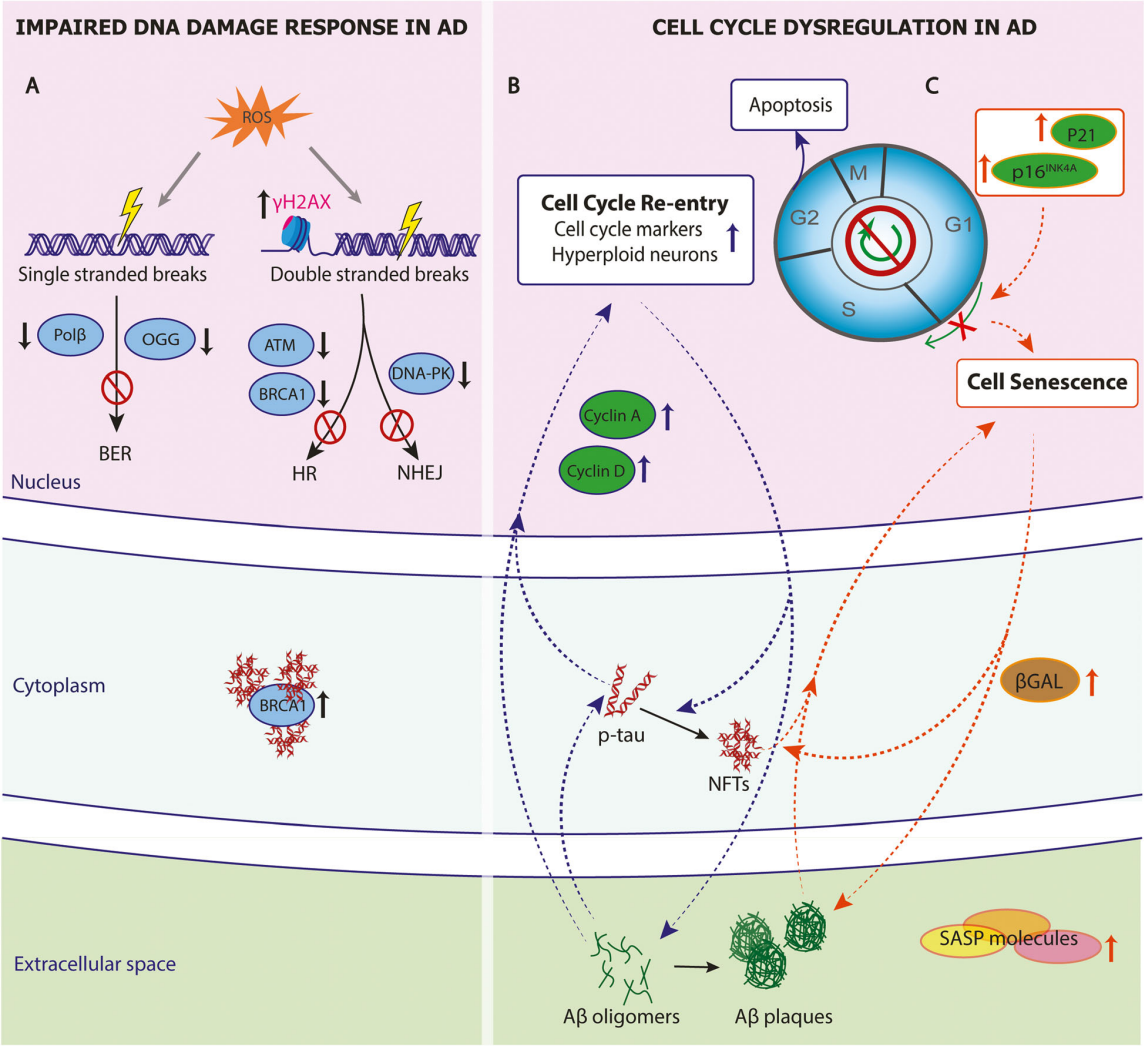
DNA damage and cell cycle dysregulation in AD. **A** Reactive oxygen species (ROS) cause DNA single- or double-stranded DNA breaks in AD. The histone variant H2AX (γH2AX), a marker of DNA double breaks, is increased. The enzymes and pivotal molecules for base-excision pair (BER), homologous recombination (HR), and non-homologous end-joining (NHEJ) repairing pathways are reduced, leading to reduced DNA damage response in AD. BRCA1, a pivotal molecule for HR, is downregulated in the nuclei but increased in the cytosol, interacting with neurofibrillary tangles (NFTs). **B** Dysregulation of cell cycle regulators result in cell cycle reentry (blue labeling) or **C** cell senescence (orange labeling) in AD. Soluble forms of Aβ and tau increase cyclin A and cyclin D, leading to cell cycle reentry and cell apoptosis. The upregulation of P16, P21 likely induces cell senescence. Senescent cells also express SA-βGal and release pro-inflammatory, senescence-associated secretory phenotype (SASP) molecules. The CCR and cell senescence are likely to form feedback loops with AD pathology. Notably, Aβ oligomers and phosphorylated tau (p-tau) in their soluble forms lead to cell cycle reentry

Histone acetylation changes have also been implicated in AD pathogenesis (Fig. 2A). Early work from the Johnson group reported elevated expression of HDAC6 in human AD cortices and hippocampi [39]. Interestingly, they showed that HDAC6 interacted with tau, and inhibition of HDAC6 in HEK cells did not disrupt HDAC6-tau interaction but attenuated tau phosphorylation [39]. Tsai group conducted ChIP-PCR on the hippocampal CA1 tissue of the CK-p25 AD mouse model and revealed loss of H2BK5ac, H3K14ac, H4K5ac, and H4K12ac on neuroplasticity genes. They further experimentally validated that this epigenetic blockade was mediated by elevated HDAC2, which was also detected in the CA1 area of 5XFAD mice and in AD patients (Braak I–VI) [40]. The initial effort of using targeted proteomics to measure histone acetylation was made to measure H3K18/K23ac in a limited set of human samples and found a significant reduction of H3K18/K23ac levels in the AD temporal cortex [41]. A recent study demonstrated that astrocytic ApoE particles promote acetylation of H3K9, H3K27, H4K5, and H4K12 in cultured neurons, which subsequently enhanced transcription of neuronal immediate early genes (IEGs) that favor memory consolidation [42]. Indeed, ApoE knockout mice showed drastically reduced H3K27ac marks on the promoter regions of IEGs in response to a learning and memory training paradigm, and human ApoE4 targeted replacement (TR) mice demonstrated less enriched H3K27ac than ApoE3 TR mice, indicating that ApoE4 is less capable of promoting histone acetylation [42]. In line with those studies, HDAC inhibitors have shown promise as a therapeutic approach to combat the cognitive impairment associated with aging and neurodegenerative disease [43–45].

The application of ChIP-seq has enabled genome-wide analysis of acetylation patterns in postmortem AD brain tissue. In this regard, Nativio et al. conducted the first ChIP-seq for H4K16ac, a key modification related to aging and cellular senescence [46], on the lateral temporal lobe of 31 younger and elderly cognitively normal controls as well as AD patients. They found that H4K16ac peaks were predominantly increased with normal aging but lost in AD. Notably, altered H4K16ac peaks in the AD cortex were enriched for AD-associated single nucleotide polymorphisms (SNPs) and expression quantitative trait loci (eQTL) [46]. Recently, the Mill group conducted ChIP-seq for H3K27ac, a robust mark of active enhancers and promoters, on the entorhinal cortex of 47 elderly individuals comprising of both AD cases (Braak VI) and controls. They identified thousands of differential peaks in AD brains associated with transcriptional alterations at nearby genes [47]. Consistent with the H4K16ac study, those H3K27ac differential peaks also represented a significant enrichment of AD risk variants, including genetic regions involved in AD neuropathology such as *APP*, *PSEN1*, *PSEN2*, and *MAPT*. With a sample size of 669 cases from the ROSMAP cohort, Klein et al. conducted ChIP-seq for H3K9ac, another histone mark for transcriptionally active open chromatin, in the dorsolateral prefrontal cortex of control and AD individuals. They found that tau protein burden, but not Aβ, coincided with widespread H3K9ac chromatin remodeling, and the majority of H3K9ac domains resided in the open chromatin region and were positively correlated with transcriptional changes in AD brains [48].

#### DNA methylation in AD

The most abundant and broadly studied DNA modification, 5-methylcytosine (5mC), is the addition of a methyl group at the cytosine in a CpG dinucleotide. Another stable epigenetic mark that is abundant in the brain is 5-hydroxymethylcytosine (5hmC), an oxidized form of the canonical 5mC catalyzed by ten-eleven translocation (TET) enzymes [49]. Although less prevalent, non-CpG (CpH) methylation also plays a critical role in many biological processes. It is widely accepted that increased methylation in promoter regions results in transcriptional repression, whereas hydroxymethylation of the same loci is associated with transcriptional activation [50].

Global methylation was initially assessed by immunohistochemistry but with inconclusive results. Mastroeni et al. first reported significantly reduced immunoreactivity of 5mC in tangle-bearing neurons of the temporal cortex of AD individuals [51, 52]. However, results from other studies using similar antibody-based methods showed either increased [53–55] or unaltered DNA methylation in the AD cortex [54]. With the advent of bead-based methylation arrays, extensive genome-wide profiling of DNA methylation was conducted in multiple brain regions of individuals with AD. Much like the immunohistochemistry results from global DNA methylation studies, the results have been inconclusive. Nevertheless, these methylation-wide association studies (MWAS) have revealed common methylation changes at a number of AD risk loci such as *ANK1*, *BIN1*, *RHBDF2*, *HOXA3*, *CDH23,* and *RPL13* [56–61], providing relatively strong evidence that methylation of these genetic loci may be altered in AD. Recently, Zhang et al. conducted a meta-analysis of more than 1000 prefrontal cortex brain samples and identified 119 differentially methylated loci significantly associated with Braak stage progression; the most significant locus is the *MAMSTR* gene, a cofactor that regulates PU.1, a central gene hub in the AD [57]. Furthermore, Smith et al. combined the data of three cortical regions from six independent AD MWAS and identified 220 differentially methylated CpGs associated with the Braak stage, provided additional significant new differentially methylated loci, including *PPT2/PRRT1*, *AGAP2*, *SLC44A2,* and *ADAM10* [62].

The majority of published AD MWAS studies are performed in bulk brain tissue that contains multiple cell types, including neurons, astrocytes, and microglia, all with potential distinct methylation patterns. Although corrections for cell-type composition through reference-based algorithms are applied, establishing cell-type-specific methylation changes is still challenging due to the different ratios between neurons and glia across brain regions. An exciting improvement in recent studies is to conduct methylation assays on enriched cell types isolated by fluorescence-activated cell sorting or laser-assisted microdissection [63–66]. Based on these studies, neurons and astrocytes each demonstrate thousands of differentially methylated CpGs associated with Braak stages but with only ~ 5% overlapping. Glia, especially microglia, primarily exhibit prominent CpG methylation in the *ANK1* gene, whereas CpG methylation in neurons occurs in the *BIN1*, *SEC14L1*, *BRCA*, and *MCF2L* genes [63–66]. Differentially methylated sites in AD neurons are primarily hypomethylated at CpH sites in the enhancer regions, associated with upregulated ∝-secretase 1 and increased plaque and tangle formation [67].

Association studies of 5hmC with AD neuropathology have been sprouting in the past couple of years. Coppieters et al. reported a global increase of both 5mC and 5hmC in neurons (but not glia) of AD frontal and temporal cortex using immunohistochemistry, correlated with AD pathology load [53]. While still lacking power and sample size for meta-analyses, some interesting findings have emerged. In postmortem AD brains, locus-specific changes in 5hmC have been associated with AD pathology [68]. By simultaneously profiling 5mC and 5hmC levels, Smith et al. discovered hypermethylation and hypohydroxymethylation at the *ANK1* promoter in AD brains [69]. Recently, Lardenoije et al. revealed a novel differentially hydroxymethylated region in the *CHRNB1* gene that encodes acetylcholine receptor beta subunit, crucial for cholinergic neurotransmission [60]. Moreover, Zhao et al. performed 5hmC-capture sequencing and identified various differentially hydroxymethylated regions associated with plaques or neurofibrillary tangles. They also developed differential co-methylation network analysis and identified various modules with unique hub genes that drive AD pathology [68].

#### Enhancers in AD

Enhancers are gene regulatory elements where transcription factors bind to influence spatiotemporal gene expression programs [70]. Enhancers can undergo three-dimensional interactions with promoters either locally or over large distances to regulate gene transcription [71–73]. In addition, enhancers often show tissue- and cell-type-specific activities [74–76], and neuronal enhancers are also regulated by cell activities [77].

SNPs in enhancer regions can influence the expression of genes and predispose individuals to AD [78]. Gjoneska et al. profiled seven chromatin states and transcriptional changes during the pathological progression of the hippocampus in the CK-p25 AD mouse model. They mapped orthologous genes in noncoding regions between mouse and human and found strong conservation of gene expression and epigenomic signatures. Notably, AD-associated SNPs were specifically enriched in increased-level enhancer orthologues with immune function, implicating immune processes in AD predisposition [79]. By integrating AD SNPs with publicly available data for enhancers that were annotated from 127 human tissues or cell types, a recent study revealed that about 96% of AD SNPs localize in non-coding regions, and 27% in enhancers [80]. Among those enhancer SNPs, 95% reside in the same topological associated domains with their eQTL genes and genes associated with synaptic transmission, immune responses, and Aβ metabolism [78, 80, 81].

Although the field just started to understand how enhancer variants affect gene expression in AD, some exciting studies have emerged. For instance, some AD enhancer variants regulate multiple eQTL genes by affecting the binding of CTCF or other cohesin complex subunits and chromatin looping [80]. The rs7364180 AD variant alters the expression of the transcription factor SREFB2 and then indirectly regulates 20 AD risk genes through a cascade of transcriptional events [80]. The CLU intron variant rs2279590 affects *CLU* expression and two other AD risk genes *EPHX2* and *PTK2B*, by eliminating a transcription factor binding site for heat shock factor 1 (HSF1) [82]. Since most enhancers are unique to specific cell types, AD enhancer SNPs likely confer their functions in a cell-type-specific manner [83]. A powerful method to map active promotor-enhancer interactome in specific cell types is to utilize proximity ligation-assisted ChIP-seq (PLAC-seq) in which proximity ligation preceded an enrichment for active promoters by H3K4me3 ChIP-seq. Using this approach, Nott et al. identified AD candidate causal variants in microglia-specific enhancers that were looped to corresponding active promoters. Indeed, deletion of a BIN1 microglia-specific enhancer harboring AD-risk variants ablated *BIN1* expression in iPSC-derived microglia but not in neurons or astrocytes [81].

### Nuclear stress responses in AD

#### DNA damage response

DNA lesions are sites of damage in the base-pairing or structure of DNA, classified as single-strand breaks (SSBs) and double-strand breaks (DSBs). They occur as either physiological or pathological cellular processes. Nevertheless, cells often initiate various mechanisms, termed DNA-damage response (DDR), to recognize and repair these incidents. Specifically, SSBs are usually recognized and corrected by the base excision repair (BER), and DSBs by either the error-prone non-homologous end-joining (NHEJ) or the homologous recombination (HR). If the damage remains unrepaired, genome instability, cellular senescence, and cell death can subsequently occur [84, 85] .

In AD, multiple brain cell types have been reported to harbor DNA damage due to oxidative stress and the inefficient DDR [85]. Evidence for DNA aberrations dates back to 1999, when DSBs and SSBs were detected in hippocampi of AD brains [86]. More recently, studies also showed increased levels of γH2AX, a well-established marker of DSBs, in neurons and astrocytes of AD hippocampi and cortices [87, 88] (Fig. 2A). Interestingly, the elevation of γH2AX expression was detected in brains with MCI and preclinical AD, suggesting an early contribution of DNA damage to AD pathophysiology [88]. As endogenous reactive oxygen species are the major source of DNA damage, cerebrospinal fluid (CSF) levels of DNA oxidation marker, the 8-OHdG has been proposed as a biomarker for AD early diagnosis in multiple studies [89].

DNA BER pathway is the primary pathway to repair oxidized bases and subsequent DNA SSBs. In general, DNA glycosylase recognizes and removes the oxidized base, and then APE1 endonuclease, PNK kinase, DNA polymerase β (Polβ), and ligases III/I complete the repair. The major enzymes involved in BER have been found downregulated in AD [85] (Fig. 2A), and their changes correlate with the clinical manifestations and AD CSF biomarkers [90]. For instance, MCI and AD brains show decreased levels of Polβ, a DNA polymerase primarily responsible for replacing single nucleotides during BER [91]. Interestingly, a recent study has shown that loss of Polβ is enough to drive cells into senescence [92], another potential mechanism contributing to AD pathophysiology (see cell senescence section). In the brains of both AD and MCI patients, there is a significant reduction of 8-oxoguanine DNA glycosylase (OGG), which excises oxidized DNA thereby preventing its accumulation [91]. Therefore, the profound changes of OGG, and Polβ at the MCI stage suggest that the impaired BER responses could occur before overt AD pathology.

Poly (ADP-ribose) polymerase 1 (PARP1) also contributes to BER by detecting an SSB and then signaling other DNA-repairing enzymes. In AD brains, elevated levels of poly (ADP-ribosylated) proteins, products synthesized by PARP1, have been detected [93]. Furthermore, DNA damage caused by Aβ can activate PARP-1 in astrocytes, dopaminergic neurons, and hippocampal slices, which can further induce the p53 and reduce the Bcl-2 protein expression, leading to cell apoptosis [94, 95].

DSB repair pathways mediated by HR or NHEJ are also involved in AD (Fig. 2A). For instance, ATM and BRCA1, two pivotal molecules for HR, have been found downregulated in AD brains and iPSC-derived neurons [96, 97]. A postmortem neuron-specific DNA methylome study revealed that the *BRCA1* promoter was hypomethylated in AD, accompanied by a reduced BRCA1 expression in the nuclei but an increased expression in the cytosol, especially in tau-bearing insoluble aggregates [65]. Likewise, literature also suggests a compromised NHEJ-mediated repair pathway in AD. First, end-joining activity and protein levels of DNA-dependent protein kinase (DNA-PK), a kinase involved in repairing DSBs through NHEJ, were found reduced in AD cortices [98]. Moreover, the MRE11, a protein complex essential for NHEJ responses, is also decreased in AD cortical neurons [99].

#### Cell cycle deregulation

##### Cell cycle re-entry

In eukaryotes, the cell cycle consists of four discrete phases: G1, S, G2, and M. Progression through these phases is regulated by cyclin-dependent kinases (CDKs) [100]. Neurons in the adult brain are terminally differentiated and generally thought to be incapable of re-entering the cell cycle. However, multiple studies suggest that neurons can re-enter the cell cycle from their quiescence G0 to G1 phase upon cellular stress and then continue into S, G2, or M phase [101–106]. However, only a small number of those neurons eventually divide [106], and most of them undergo apoptosis [107]. Cell cycle re-entry (CCR) is likely mediated by multiple signaling pathways [104, 108–112].

Accumulated studies have detected cell cycle markers and regulatory proteins in postmortem brain tissue, supporting CCR present across multiple brain regions in all AD stages [113, 114] (Fig. 2B). As a result of CCR, hyperploid neurons are drastically increased at preclinical stages of AD, indicating CCR is a potentially causal event in AD pathogenesis [115]. Indeed, SV40 large T antigen-induced CCR was reported to cause cortical deposition of Aβ plaques and NFT pathology [116], in addition to neuronal degeneration [117]. Similarly, CCR induced by c-myc and ras oncogenes also increases p-tau levels in cultured primary cortical neurons [118]. Furthermore, overexpressing denticle-less (DTL), a potent cell cycle regulator, induces CCR and subsequent tau hyperphosphorylation, Aβ production, and cognitive impairment in mice [119]. Consistently, many other studies also demonstrate that CDKs can drive the Aβ plaque formation [120–123] and tau phosphorylation [124, 125] (Fig. 2B).

On the other end, research evidence also shows that AD pathology triggers neuronal CCR. First, knock-in mice harboring human *APP* and *PSEN1* show increased cyclin A and cyclin D1 in hippocampal and cortical neurons, leading to CCR and cell apoptosis [103, 126]. Moreover, oligomeric Aβ induces dose-dependent neuronal CCR, driving neurons into different cell cycle phases or apoptosis [111, 127]. This Aβ-induced CCR depends on tau phosphorylation by multiple protein kinases activated by Aβ, indicating soluble forms of Aβ and tau are the essential elements for CCR [128]. Genetically perturbing cell-cycle progression in tau-expressing *Drosophila* models can reduce tau-induced neuronal apoptosis [129]. As such, Aβ oligomers, phosphorylated tau, and CCR are likely to form feedback loops at the early stage of AD and ultimately lead to neuronal apoptosis (Fig. 2B).

##### Cell senescence

Senescence is an irreversible cell cycle arrest due to the blockade to the S phase of the cycle. Cell senescence related to aging and neurodegeneration is often chronic. It includes replicative senescence [130], stress-induced premature senescence [131], and mitochondrial dysfunction-associated senescence [132]. Despite different categories, chronic cell senescence is generally characterized by a proinflammatory senescence-associated secretory phenotype (SASP), altered mitochondrial function, cellular metabolism, and DNA damage [133, 134].

Cell types in the central nervous system, including neurons, astrocytes, microglia, oligodendrocytes, have been reported to undergo cell senescence during aging. In AD, Aβ plaques and NFTs, along with other cellular stressors, have been shown to induce DNA damage and alter chromatin structure, and subsequently leading to cell senescence [36, 135–143] (Fig. 2C). In postmortem AD brains, Aβ plaques are commonly associated with oligodendrocyte precursor cells expressing senescent markers SA-βGal, p21 (CDKN1A), and p16^INK4^(CDKN2A) [144] (Fig. 2C). Moreover, laser-dissected neurons from AD brains also bear a transcriptomic profile characteristic of cell senescence, including proinflammatory cytokines and senescence-related upstream regulators [141] (Fig. 2C). Furthermore, senescent astrocytes marked by p16^INK4A^ and MMP-1 are increased with age and were more prominent in age-matched AD cortices [145]. Finally, microglia with dystrophic morphology and shorter telomeres also increases with age, but with a significantly higher number in AD brains [146–148]. Interestingly, a recent study indicates that increased myelin breakdown with age overwhelms microglial phagocytosis function, contributing to microglial senescence [149].

Mouse studies also provided evidence of senescent glia and neurons near Aβ plaques or NFTs, but the cell types undergoing senescence varied among animal models [141, 144, 145, 150, 151]. However, regardless of cell types affected, ablation of senescent cells using either chemical or genetic approaches was protective against AD progression, indicating that cell senescence causally contributes to AD pathogenesis (Fig. 2C). For instance, selectively ablating senescent cells by senolytics in AD mouse models reduces SASP, neuroinflammation, plaque size, NFT burden, and alleviates cognitive declines [141, 144, 151]. Because senescent cells undergo profound chromatin and gene alterations [152–154], perturbing related factors have been shown to alter cell senescence and AD phenotype. For example, mice lacking one allele of *Bmi1,* a core component of the polycomb repressive complex, shows relaxed heterochromatin, cellular senescence, amyloid plaque, and p-tau formation; meanwhile, introducing mutant APP to *Bmi1*-deficient mice exacerbates amyloid and tau pathology [155].

The fate choice for stressed cells toward CCR or cell senescence is yet to be investigated in AD. Stressors such as oxidative stress, neuroinflammation, hypoxia, and DNA damage can affect nuclear integrity and regulation, inducing CCR or cell senescence [156–160]. Recent studies suggest that senescent cells result from insoluble NFT formation [141, 151], whereas CCR occurs before NFT and plaque formation [128], indicating the solubility of AD-related proteins as a potential determinant toward cell senescence or CCR. Thus, understanding the molecular underpinning of cell senescence and CCR will help us develop therapeutic strategies to mitigate cell cycle dysregulations in AD development.

## Conclusions

This review discussed nuclear dynamics and nuclear stress response in AD, focusing on nuclear architecture, chromatin modifications, and nuclear stress responses. These nuclear characteristics are dynamically regulated to collectively maintain cellular homeostasis. Therefore, abnormal changes reviewed here present the major nuclear perspectives of the AD pathological process. While this review focuses on nuclear mechanisms, multiple gene regulations outside the scope of this review have also shown emerging evidence of their implications in AD, including transcriptional factors [161–163], RNA splicing [164–166], RNA editing [167], RNA binding proteins [168], microRNAs [169], nuclear non-coding RNAs (ncRNAs) [170], and enhancer RNAs (eRNAs) [170, 171]. These gene regulators have been shown to shape gene expression and modulate chromatin architecture [170], but their precise mechanisms in AD remain to be explored [172, 173].

It is worth noting that molecular changes in AD often intertwine and occur concurrently to mediate AD progression. For instance, lamin dysfunction in a tau-transgenic fly model of AD leads to heterochromatin relaxation and DDR [6]. DDR is also reported to trigger CCR and cell senescence [174–177] and reduces nucleolar rRNA transcription [31, 34]. Multi-omics studies from AD mouse models and postmortem brain tissue showed concordant changes among chromatin states, DNA accessibility, transcriptomics [48, 79], and widespread loss of CpH methylation at enhancers of AD neurons significantly converge on transcriptomic changes related to abnormal CCR, apoptotic and inflammatory pathways [67]. Furthermore, SNPs influencing epigenomic marks (xQTLs) overlap significantly with splicing QTLs in AD, and there is significant sharing of xQTL SNPs across the AD molecular phenotypes [165, 178]. Repressor element 1-silencing transcription factor (REST), mediates active epigenetic repression of many genes that promote cell death and AD pathology, and at the same time, induces the expression of stress response genes [179].

How the different nuclear regulations occur coherently within the nucleus is still not clear. An emerging concept is that most nuclear regulatory processes occur through dynamic nuclear condensates that compartmentalize regulatory proteins and RNA molecules to proper genomic loci for coordinated nuclear regulations [180, 181]. The way the condensates are involved in disease progress is yet to be investigated. Notably, a recent study has revealed that the causal mutation of the methyl CpG binding protein 2 (MeCP2) disrupts its ability to form heterochromatin condensates, suggesting a novel mechanism for Rett syndrome [182].

One major challenge in AD research is understanding cell-type-specific molecular changes and their responses to intra- and extracellular pathology. We have seen exciting advances in applying single-cell biology and spatial transcriptomics in AD postmortem tissue and animal models in the past couple of years. These studies have provided invaluable information on cell-type-specific transcriptomic changes and revealed cell types implicated in early AD [183–186]. Furthermore, single-soma transcriptomics of tangle-bearing neurons directly maps tangle pathology to gene changes, proving an exciting approach to understanding pathology heterogeneity of single neurons in AD [186]. Lastly, a recent spatial transcriptomics study provides the first spatial map of transcriptional changes in the vicinity of AD pathogenic hallmarks and identifies plaques-induced gene networks in the early and late AD phases, respectively [183]. With these technologies rapidly evolving, we expect to see more impressive research systematically mapping multidimensional molecular changes in AD with unprecedented cellular, spatial and temporal resolution.

Another challenge is that epigenetic and transcriptomic changes in AD could result from genetic variants or/and pathological insults. Therefore, identifying the causal genetic variants and variant-driven transcriptional changes will allow us to construct the genetic circuitry of AD pathogenesis, thereby providing a better strategy for early AD intervention. By combining CRISPR gene editing with iPSC-based cell models, numerous studies have provided significant insights into the role of genetic variants in AD development [187–189]. Genetic perturbations can also be implemented in massively parallel genetic screens to interrogate gene functions in iPSC-derived neural cell types [190]. Elegant genetic screening studies have been conducted in iPSC-derived neurons to identify causal genes for cell survival, oxidative stress, and lysosome dynamics [191, 192]. The advent of base editors [192, 193] and prime editing [194] technologies enable all possible single-base transition and transversion, providing a powerful platform to interrogate the function of genetic variants in AD development and establish the causal links from genetics to various intermediate molecular phenotypes.

Lastly, the dynamic nuclear structure alterations that contribute to AD can be investigated using experimental and computational approaches developed by the 4D nucleome project [195]. Visualizing chromatin contact sites with super-resolution microscopy [196] or sequencing [197, 198] has started to reveal exciting insight into chromatin structure changes in AD [199]. Implementation of these state-of-the-art technologies will help explain how the nuclear genome is maintained and regulated in AD progression, providing novel mechanistic insights into the molecular events and their dynamic progression.

## Data Availability

Not applicable.

## Abbreviations

5hmC: 5-hydroxymethylcytosine
5mC: 5-methylcytosine
Aβ: Amyloid-beta
AD: Alzheimer’s disease
BER: Base excision repair
CCR: Cell cycle re-entry
CDKs: Cyclin-dependent kinases
ChIP-seq: Chromatin immunoprecipitation followed by sequencing
CSF: Cerebrospinal fluid
DDR: DNA-damage response
DNA-PK: DNA-dependent protein kinase
DSBs: Double-strand breaks
DTL: Denticle-less
eRNA: Enhancer RNA
eQTL: Expression quantitative trait loci
HATs: Histone acetyltransferases
HDACs: Histone deacetylases
HP1α: Heterochromatin protein 1α
HR: Homologous recombination
HSF1: Heat shock factor 1
LADs: Lamina-associated domains
lncRNA: Long noncoding RNA
LoNA: Long nucleolus-specific lncRNA
MAPT: Methyl CpG binding protein 2
MCI: Mild cognitive impairment
MWAS: Methylation-wide association studies
ncRNAs: Non-coding RNAs
NFTs: Neurofibrillary tangles
NHEJ: Non-homologous end-joining
NPC: Nuclear pore complex
NTF2: Nuclear transport factor 2
Nups: Nucleoporin proteins
OGG: 8-oxoguanine DNA glycosylase
PLAC-seq: Proximity ligation-assisted ChIP-seq
Polβ: DNA polymerase β
PPARγ: Peroxisome proliferator-activated receptor γ
p-tau: Phosphorylated tau
PTMs: Post-translational modifications
rDNA: Ribosomal DNA
REST: Repressor element 1-silencing transcription factor
RNP: Ribosomal ribonucleoprotein
rRNA: Ribosomal RNA
SASP: Senescence-associated secretory phenotype
SNPs: Single-nucleotide polymorphisms
SSBs: Single-strand breaks
TET: Ten-eleven translocation
